# Examining the potential of VR program Tilt Brush in reducing anxiety

**DOI:** 10.1007/s10055-022-00711-w

**Published:** 2022-11-10

**Authors:** Janice Tan, Lee Kannis-Dymand, Christian Jones

**Affiliations:** 1 School of Health and Behavioural Sciences, University of the Sunshine Coast, Maroochydore, QLD Australia; 2 School of Law and Society, University of the Sunshine Coast, Maroochydore, QLD Australia

**Keywords:** Virtual reality, Flow, Presence, Anxiety, Tilt Brush, Drawing

## Abstract

Recent advancement in technology has made virtual reality (VR) more accessible and immersive than ever before, resulting in its increasing utility in various industries. Despite this, VR has remained an underutilised tool within clinical psychology. This study aimed to explore the potential of using VR for therapeutic benefits through examining the level of flow and anxiety-reducing effects of freeform drawing in real life (on paper) versus drawing in VR (using Tilt Brush) via a randomised-controlled trial with 40 participants. State and trait anxiety was measured using the State-Trait Anxiety Inventory, level of flow was measured using the Long Flow State Scale, and level of presence was measured using the iGroup Presence Questionnaire. Overall level of flow was not significantly different between both groups, implying drawing in VR induces as much flow as drawing in real life. Level of flow was positively correlated to level of presence experienced in the VR group (*p* < .01). Although there was no significant interaction effect, both groups experienced an overall decrease in state anxiety, with the VR group experiencing a significant reduction of state anxiety from pre- to post-test (*p* < .01).

## Introduction

VR is a technological interface that creates simulated, computer-generated environments that allows an interactive user experience. Current VR systems (e.g. Oculus Quest, Windows Mixed Reality, Valve Index, HTC Vive) typically require users to wear a head-mounted display (HMD) which presents the audio-visual simulation of realistic or fictional environments. Over the past decade, VR has become a promising tool for scientific investigation (Snoswell and Snoswell 2019). Its capacity to simulate different realities and experiences has prompted researchers to examine the potential of incorporating VR into various medical (Li et al. 2017) and psychological (Maples-Keller et al. 2017) interventions. VR techniques have been implemented in the treatment of phobias (Botella et al. 2017), post-traumatic stress disorder (PTSD; Kothgassner et al. 2019), anxiety disorders (Chan et al. 2021; Rothbaum et al. 2002; Parsons and Rizzo 2008; Eijlers et al. 2019), depression (Falconer et al. 2016), schizophrenia (Bisso et al. 2020), eating disorders (Riva et al. 2016a, b) and pain management (Ferraz-Torres et al. 2022; Phelan et al. 2021a, b; Matamala-Gomez et al. 2019; Spiegel et al. 2019; Vilalta-Abella et al. 2015). Reducing anxiety using VR to deliver interventions has been explored and found effective through nature-based mindfulness experiences (Tarrant et al. 2018; Chan et al. 2021; Liszio and Masuch 2019; White et al. 2018), VR distraction for pain during blood drawing (Piskorz and Czub 2018; Gerceker et al. 2018; Gold and Mahrer 2018) and before entering an operating theatre (Ryu et al. 2017; Arpaia et al. 2022), and for cognitive behaviour therapy (CBT) and Virtual reality exposure therapy (VRET) (Baghaei et al. 2021). Recent technological advances have allowed the proliferation of VR technologies from specialist laboratories to widespread consumer applications, increasing the availability of such systems, and enhancing the possibilities of their use for therapy (Rowland et al. 2021; Bohil et al. 2011) as well as entertainment and art (Hacmun et al. 2018, 2021; Carrozzino and Bergamasco 2010).

### VR and art

In addition to clinical applications, VR allows for unique expressions of creativity and the extension of classical creative expressions such as painting and sculpting with the introduction of novel artistic programs (e.g. Oculus Quill, Oculus Medium, Google Blocks). This has given rise to perspectives on the potential therapeutic benefits that VR can bring in the form of creative expression (Hacmun et al. 2018). While there have been favourable reviews published on the feasibility of using VR in replacement of real-life artistic expression, there is a lack of implementation of the research. Two small sample pilot studies using Tilt Brush found that 20 min VR art-marking resulted in positive emotions (Kaimal et al. 2020a, b), and 2 × 1 h VR art-making (with and without an airborne fragrance) showed a reduction in stress (Kaimal et al. 2020a, b). A study comparing 15 min of 3D VR Tilt Brush to 2D art making found a reduction in measures of HR, negative affect, state anxiety and trait anxiety, but reports its limitation in omitting measures of presence or flow (Richesin et al. 2021). Hence, this study aimed to explore how creative mediums provided by VR can be potentially employed for anxiolytic purposes.

### Art and anxiety reduction

Creating visual art is often described as relaxing or calming. Creative activities have been found to reduce anger and anxiety in women with stage I and II breast cancer (Puig et al. 2006), as well as anxiety and negative mood in undergraduate students (Drake et al. 2014). Sandmire et al. (2012) observed that a brief 30-min period of art-making significantly reduced anxiety in a sample of undergraduate students who were due to sit for their final examinations the following week. Laurer and van der Vennet (2015) sought to examine if the anxiety-reducing effect of art would differ if it was being viewed and sorted, rather than produced. They found the act of producing art resulted in a reduction of negative mood and anxiety, compared to simply looking at art. A systematic review and meta-analysis of eight studies investigating the effect of free drawing found all reduced state anxiety after a single, short drawing session, with the length of time drawing ranging between 10 and 20 min (Jakobsson Støre and Jakobsson 2022).

While creative activities have demonstrated anxiety reducing capabilities, the mechanism behind this has not been well-studied. In a study by Belkofer et al. (2014), a significant increase in alpha brainwave levels was observed in participants after 20 min of drawing. Alpha brainwaves are associated with relaxed states of consciousness (Swingle 2008), giftedness (Jausovec 1996), and divergent thinking (Fink et al. 2006) and has been found to play a role in meditation (Cahn and Polich 2006; Lagopoulos et al. 2009), visualisation, working memory, and self-regulation (Knyazev 2007; White and Richards 2009). In more recent publications, alpha brainwaves are also associated with mindfulness (Lomas et al. 2015) and flow (Katahira et al. 2018). Findings from other studies also seem to point in the direction of flow as a mechanism for anxiety reduction in creative activities. Reynolds and Prior (2006) conducted a qualitative study on female cancer patients who engaged in visual art making to reflect on their experiences of the creative process. Participants described numerous experiences associated with flow such as experiences of mastery and control, evolving imagery, and creative adventures. A recent study also supported the association between art-making and flow, along with increased ability to feel pleasure and inspiration (Holt 2018). While a wide body of research has suggested large treatment effects for VR exposure-based therapy for anxiety disorders (Bandelow et al. 2015), none have examined the use of VR in art marking to reduce anxiety through mindfulness and flow. Hence, this study aimed to contribute to this gap in the existing literature.

### Flow

Flow involves being fully consumed with the present moment and with the task at hand (Csikszentmihalyi 1992). Also known as “effortless attention”, flow is a total immersion into an activity that results in the combination of action and awareness, centring of attention, and the loss of self-consciousness whilst still maintaining a feeling of control. Flow is a psychological construct that can be described as an optimal experience in which an individual is fully engaged within an activity, and can be experienced during the creation of art.

While neuroscience research has shown that there is a great deal of mental activity involved in the creation of art (Chávez-Eakle et al. 2007), the experience of flow in art making can feel effortless. Yet, task performance in flow has been shown to be better than non-flow (Dietrich 2004). The transient hypofrontality theory explains this with two fundamental neuroscience principles: the brain has finite energy supply and that information processing in the brain is based on competitive interactions among neurons (Dietrich 2004; Dietrich and Audiffren 2011; Dietrich and Stoll 2010). The brain in flow activates two information systems: the explicit information system for higher-order cognitive processing and conscious awareness, and the implicit information system for skill-based knowledge not available to verbal processing (Dietrich 2004). According to Dietrich and Audiffren (2011), “tasks that require real-time sensorimotor integration are best handled by the implicit system” (p. 1308), such as various creative activities such as creating visual art and drawing. Participants from previous studies involving dancers (Hefferon and Ollis 2006) and athletes (Jackson 1996) have reported experiencing flow as distorted sense of time, lack of worry, reduced awareness of the self, and effortless attention. Therefore, when flow state is attained, energy for the explicit system shifts to the implicit system, resulting in the characteristics that define the experience of flow (Dietrich 2004).

### Presence

It is projected that the consumer market of VR hardware and software will leap from 6.2 billion US dollars in 2019 to over 16 billion US dollars by 2022 (Alsop 2022). What makes VR so attractive and impactful is its ability to induce a feeling of “presence” in the computer-generated world experienced by the user (Riva et al. 2016a, b). VR aims to parallel reality and create a world that is both immersive and interactive (Rizzo et al. 1997). VR allows the creation of a subjective experience, giving its user the illusion that the experience is real via mimicking the sensory (i.e. visual, auditory) and motor (e.g. immersive environment, motion tracking) signals and contingencies found in the real world. It has been suggested that this sense of presence can serve as a powerful therapeutic tool for promoting personal change and self-reflectiveness (Riva et al. 2016a, b).

Built on Damasio’s theory of consciousness (1999), presence can be divided into 3 layers: proto presence, core presence, and extended presence. Each layer accounts for a facet of the internal/external world and is characterised by specific properties (Riva et al. 2004). Proto presence is the sense of physical being and other ways of knowing bodily orientation in the world. Virtually known as “spatial presence”, this requires the tracking of physical body parts while updating the displays in sync. Core presence is the activity of selective attention made by individual perception. In the virtual world, this is known as “sensory presence” and requires good quality graphics and displays features, preferably stereographic. Finally, extended presence can be characterised as the significance to the self of the events experienced in the external world. These three layers work together to produce different levels of presence. VR is theorised to be able to activate all 3 layers of presence (Riva et al. 2004), thereby providing the highest level of presence and promoting a level of flow that is akin to doing the activity in real life. This is further supported by previous research that found a positive association between presence and flow when participants engaged in computer and console games, such as Music Paint Machine (Nijs et al. 2012), Neverwinter Nights, Formula 1, Sonic the Hedgehog (Weibel and Wissmath 2011), Trauma Center: New Blood, Need for Speed, and The Godfather (Jin 2011), player empathy from serious games (Bachen et al. 2016; Han et al. 2022), and in VR storytelling (Yang and Zhang 2022). These computer and console games are screen-based gaming which provide simulated environment just like VR, however are not as fully immersive as VR.

### Presence, flow and anxiety

Presence, flow and anxiety are interlinked. Presence has been found to be distinct from flow (Weibel and Wissmath 2011) but significantly correlated with flow (e.g. Jin 2011). Flow is reported to be significantly negatively correlated with anxiety (Tse et al. 2022; Mao et al. 2020), particularly music performance anxiety (Kirchner et al. 2008; Moral-Bofill et al. 2022), and anxiety and presence synergistically linked during VR immersion with phobic and non-phobic participants (Robillard et al. 2003). In the short term anxiety can cause many physical symptoms, such as shaking, dizziness, fatigue, aches, increased blood pressure, heart palpitations and breathing problems, and left unchecked can cause long-term issues such as migraines, heart disease, bowel disorders, lowered immune responses, and memory problems (Banyan Mental Health 2022). Through activating high levels of presence and inducing flow, it is possible to reduce symptoms of anxiety and their effects.

### Benefits of VR

VR allows for expression that is unrestricted by natural physical laws. For example, 3D objects can be suspended in mid-air seemingly defying gravity, creating elements whose physical properties can dynamically change over time, unrealistic and fantastic colours and textures. There is dynamic control of spatial dimension in VR; the canvas size is practically infinite, and creations can be re-scaled and re-sized when desired. Additionally, there are options to create multisensory creations such as adding music which may modify aspects of the artefact (e.g. colours). Taken together, VR allows for artistic creation that expands on classical aspects of art-making (e.g. colour palette, brushes), while adding novel features which are unique to VR (e.g. scalability, dynamic and temporal objects). These features give the user the ability to customise and create almost any environment or content according to their needs and desires.

In addition to the aspects of presence and unrestricted physical laws, VR allows for user experiences to be consistently replicated and modified within a controlled environment (Riva et al. 2004). Clinically, VR can help patients manage their psychological disturbances in a safe environment, whilst developing helpful coping strategies to overcoming their difficulties (Morganti 2004). This has been tested with positive preliminary outcomes such as increased motivation and playfulness in children with cerebral palsy (Garcia-Hernandez et al. 2021; Miller and Reid 2004), support for rehabilitation from spinal cord injury (Riva 2000), treatment of Amblyopia (Jiménez-Rodríguez et al. 2021) and increased confidence and lower anxiety when navigating a hospitalisation (Yin et al. 2012). VR interventions can be used unfacilitated (eliminating any need for a professional to commence or operate the program), low cost as evident by the availability of VR headsets for personal use, and available at any time and anywhere.

### Hypotheses

While VR is known to induce presence and promote flow (Tse et al. 2022; Mao et al. 2020), and has been used for clinical psychology in clinical cases (Robillard et al. 2003), studies comparing the effects of creative VR programs in reducing anxiety with more traditional methods of creating art are limited. Hence, this study compares the effect on anxiety between creating visual art using a VR drawing application, Tilt Brush (the experimental condition) and traditional paper and pen (the control condition), and the relationship of anxiety with presence and flow. Based on the literature review, the hypotheses of the study are:

1. Equal or greater level of flow will be experienced in the VR experimental condition than in the control condition.
2. Equal or greater reduction in state anxiety following the intervention will be reported from the VR experimental condition than from the control condition.
3. The level of flow experienced in the VR experimental condition will be positively correlated with the level of presence experienced in the VR experimental condition.

## Method

### Study design

The study was a between-subjects randomised controlled trial. The independent variable was the medium of materials used for the study’s activity. An experiment group used the VR program Tilt Brush, while the control group used physical art supplies (the following were provided to participants: colour pencils, water colours, paint, sketching pencils, oil pastels, colour markers, chalk) to draw on paper. The dependent variables were the levels of anxiety (state and trait) and level of flow felt by participants. Participants in the experiment group also reported the sense of presence felt in the VR program. Modifying the instructions used in the study by Belkofer et al. (2014), standardised instructions were used in both groups: “Use (Tilt Brush/these art supplies) to draw for 30 min. Your creation can be something abstract or concrete, and its significance is up to you. It will not be evaluated in any way”.

### Participants

Eligibility criteria for the study included: (a) Participants had to be at least 18 years old; (b) No existing mental health diagnosis; (c) No physiological condition that impacted their ability to hold a pen or VR controller. All participants were asked to bring corrective eyewear if they needed it to read or draw, resulting in normal or corrected to normal vision for participants in both groups.

A total of 40 participants (11 male, 29 female, *M*_age_ = 33.58, SD_age_ = 11.62, age range = 19–68) were recruited for this study via snowball sampling (e.g. social media, poster advertising). There were no dropouts from the study, resulting in a total of 20 participants randomly assigned to the experiment group (7 male, 13 female, *M*_age_ = 34.00, SD_age_ = 12.00, age range = 19–68 years) and 20 randomly assigned to the control group (4 male, 16 female, *M*_age_ = 33.15, SD_age_ = 11.44, age range = 22–55 years). Self-reported ethnicity was equally represented in both the experiment and control groups, comprising of *n* = 18 Caucasian and *n* = 2 Asian in each group, as was highest education level, *n* = 17 higher education and *n* = 3 secondary school. Most of the participants were university students (*n* = 33, 82.5%) from Australia (*n* = 33, 82.5%).

### Measures

#### iGroup presence questionnaire (IPQ)

The English version of the iGroup Presence Questionnaire (IPQ; Schubert et al. 1999) was used to measure presence. The IPQ is a self–report questionnaire composed of 14 statements rated on a 7-point scale on the extent they apply to oneself (varying from − 3 = fully disagree/not at all, to + 3 = fully agree/very much). The IPQ consists of three subscales: Spatial presence (SP), involvement (INV) and experienced realism (REAL). According to Schubert et al. (1999, 2001), SP measures the sense of being physically present (e.g. “Somehow I felt that the virtual world surrounded me.”), INV measures the attention devoted to the virtual environment and involvement experienced (e.g. “I was not aware of my real environment.”), and REAL measures the subjective experience of realism (e.g. “I felt like I was just perceiving pictures.”). Additionally, one general item is identified as a measure of a general sense of “being there” (PRES; “In the computer-generated world I had a sense of ‘being there.’”). The subscale reliabilities from the current study were consistent with two prior studies completed by igroup (2016a) and ranged from 0.68 to 0.85. The Cronbach’s alpha of the PRES subscale could not be determined because it consisted of only one item.

#### Long flow state scale: general (FSS)

The Long Flow State Scale: General (FSS-2-General; Jackson and Marsh 1996) was used to assess the extent of flow experienced in an event or activity. It assesses the nine key dimensions of flow described in detail by Csikszentmihalyi (1992): challenge-skill balance, action-awareness merging, clear goals, unambiguous feedback, concentration on task at hand, sense of control, loss of self-consciousness, a sense of time transformation, and autotelic experience. It has 36 items rated on a 5-point Likert scale ranging from 1 (strongly disagree) to 5 (strongly agree) and is designed for ages 12 and older. The average completion time is 10 min. The FSS-2-General has demonstrated good reliability. The FSS-2-General Cronbach’s alphas for this study were consistent with two large psychometric studies (Jackson et al. 2008; Jackson and Eklund 2002) and ranged between 0.76 and 0.92. Substantial factorial validity evidence has been published on the FSS-2-General (e.g. Jackson et al. 2008, 2001; Jackson and Eklund 2002).

#### State-trait anxiety inventory for adults (STAI)

The State-Trait Anxiety Inventory (STAI; Spielberger et al. 1983) is a 40-item self-reported questionnaire to measure both state and trait anxiety. It has been used widely in research and clinical settings, in screening for anxiety problems in college and high school students, and for evaluating the immediate and long-term outcomes of counselling, psychotherapy, drug-treatment programs, and behaviour modification. A person with state anxiety is an individual whose anxiety may fluctuate over time, is temporary, and may be triggered by external stimuli (Spielberger 1985). On the other hand, trait anxiety is a personality disposition that describes a person’s tendency to perceive situations as threatening, and hence to experience state anxiety in stressful situations (Gaudry et al. 1975). Trait anxiety is not observed directly but is expressed as state anxiety when stress is experienced (Reiss 1997, p. 204). These personality characteristics may be influenced or triggered by residues of past experiences (Spielberger 1985).

The original STAI consists of two separate scales to measure state and trait anxiety. The State-anxiety scale (STAI Form Y-1) consists of 20 items (item 1 to item 20) that measure the respondent’s feeling in that moment (i.e. state anxiety). The Trait-anxiety scale (STAI Form Y-2) also consists of 20 items (item 21 to item 40), and measures how the respondent “generally” feels (i.e. trait anxiety). All items in both Form Y-1 and Form Y-2 are rated on a 4-point scale, where state anxiety items assess intensity of current feeling (1 = not at all, 2 = somewhat, 3 = moderately so, and 4 = very much so) and trait anxiety items assess frequency of feeling in general (1 = almost never, 2 = sometimes, 3 = often, and 4 = almost always). A high score indicates the presence of high levels of anxiety. Scores for both the State Anxiety Scale and Trait Anxiety Scale ranged from a minimum of 20 to a maximum of 80.

Considerable evidence attests to the construct and concurrent validity of the scale (Spielberger 1989). Given the transitory nature of anxiety states, measures of internal consistency such as the alpha coefficient provide a more meaningful index of the reliability of State-anxiety scales than test–retest correlations. Cronbach’s alpha coefficients for the State-anxiety and Trait-anxiety scales in this study (0.85–0.95) were comparable with those reported by Spielberger et al. (1983) (0.90–0.94).

### Procedure

Participants attended a private room at the University, and first completed the demographics form and the STAI questionnaire. Following this, participants in the experiment group were instructed to stand in a play area (5.2 m × 2.6 m) consisting of the Oculus Quest headset. Participants in the control group were invited to sit at a table setup with art supplies. Prior to commencing the activity, participants were informed that they could ask any questions they had regarding the respective activities or equipment at any time during the study.

For the VR experiment, participants retrieved the VR headset and controllers from a table next to the play area and a short verbal and visual tutorial was given to participants on how to wear the headset, hold the controllers, and functions of the Tilt Brush program. Once participants demonstrated understanding of how to use the program and equipment, they were provided the standardised instructions.

For the control group, art supplies were pre-arranged on the table in a standardised manner for all participants and consisted of drawing paper (A3 and A4 sizes), water colours, paint pots, chalk, sketching pencils, eraser, fine tip pens, oil pastels, crayons, colour pencils, paint brushes, and colour markers.

After 30 min, participants were instructed to stop drawing. For the VR experiment group, they were instructed to replace the controllers and headset on the table. Participants were then asked to sit to complete the STAI and FSS questionnaires. Participants from the VR experiment group were instructed to complete an additional questionnaire, which was the IPQ. Upon completion of the questionnaires, participants were then debriefed and thanked for their time and participation. Total duration for each participant was approximately 60 min.

### Ethics approval and COVID-19 safety measures

Ethical approval was obtained from the University of the Sunshine Coast human ethics committee (approval number: S191376). As per the University of the Sunshine Coast COVID-19 restrictions and COVID-19 Safe Plan—Research, COVID-19 safety procedures were adhered to such as maintaining a social distancing of 1.5 m, sanitation of all equipment between participants, hand hygiene of researchers and participants, and COVID-19 screening questions (e.g. Do you have any flu-like symptoms? Have you returned from overseas travel (or a cruise ship) in the last 14 days?). None of the participants in this study reported feeling unwell or travelling recently.

## Results

All statistical analyses were conducted using the Statistical Package for the Social Sciences (SPSS; Version 26) program. Results were considered significant at *p* < 0.05. Assumptions were tested and met. There were no breaches of normality or heterogeneity. As multiple comparisons were performed, Bonferroni was applied to adjust for Type I errors. An estimate of the required sample size was conducted post facto using GPower (v3.1.9.4, Faul et al. 2007) with *α* = 0.05, *β* = 0.95. Within–between ANOVA analysis for anxiety required a sample size of *n* = 40 for a medium effect size of 0.3, and t-tests for independent means required a sample size of *n* = 40 for a large effect size of 1.05 (one-tailed) (GPower v3.1.9.4, Faul et al. 2007).

Independent *t*-test was performed to examine for any pre-intervention group differences. Results indicate that pre-activity State-anxiety scores were significantly different between the VR experiment and control groups (*M* = 35.90, SD = 7.11; *M* = 30.60, SD = 6.75 respectively), *t*(38) = 2.419, *p* = 0.020. There was no significant difference for Trait-anxiety between groups at pre-activity (*M* = 39.05, SD = 10.34; *M* = 38.35, SD = 11.08 respectively), *t*(38) =  − 2.07, *p* = 0.837.

Up to 30% of participants in each of the VR experiment and control groups exceeded the clinical threshold of 41 for STAI-State scores and 44 for STAI-Trait scores (Ilker et al. 2015), indicating that at some point in the study they were considered clinically anxious, Table 1. The sample size was too low to perform any statistical between-groups analysis based on clinical thresholds.

**Table 1:** Number of participants exceeding clinical thresholds for State and Trait (Ilker et al. 2015) as measured using STAI (STAI; Spielberger et al. 1983)

	VR STAI score (*n* = 20)		Control STAI score (*n* = 20)	
	Pre *n* (%)	Post *n* (%)	Pre *n* (%)	Post *n* (%)
State (> 41)	6 (30%)	2 (10%)	1 (5%)	2 (10%)
Trait (> 44)	5 (25%)	4 (20%)	5 (25%)	6 (30%)

### Presence

The mean Total IPQ score from the VR experiment group in this study was 56.95 (SD = 13.68), with comparable IPQ subscales means and standard deviations to previous studies by igroup (2016b) (Table 2).

**Table 2:** Means and Standard Deviations of IPQ scores from VR group in current study in comparison to mean IPQ scores from igroup (2016b) studies on Tomb Raider and Half Life

Dimensions	Tomb Raider	Half life	Current study
	*M*	*M*	*M* (SD)
Spatial presence	3.06	4.00	4.88 (0.90)
Involvement	2.40	3.27	4.19 (1.34)
Experienced realism	1.92	2.34	2.71 (1.40)
General	3.00	3.93	4.95 (1.15)

There was a significant large positive correlation between overall flow and total presence experienced in the VR experience group (IPQ Total), *r*(38) = 0.61, *p* < 0.01. Table 3 shows the correlation between overall flow score and the IPQ subscales (Spatial Presence, Involvement, Experienced Realism and General Sense) and IPQ total.

**Table 3:** Correlation between overall flow score and IPQ subscales and total

	Overall flow	IPQ total	Spatial presence	Involvement	Experienced realism	General sense
Overall flow	1					
IPQ total	0.61**	1				
Spatial presence	0.52*	0.81**	1			
Involvement	0.49*	0.86**	0.58**	1		
Experienced realism	0.47*	0.82**	0.46*	0.58**	1	
General sense	0.64**	0.70**	0.78**	0.52*	0.38	1

*p < 0.05 (2-tailed), **p < 0.01 (2-tailed)

### Flow

The level of overall flow in both groups were similar (VR *M* = 33.75, SD = 4.88; control *M* = 33.80, SD = 4.70). Independent t-tests were performed to compare level of flow (FSS-2-General scores) and its dimensions between both groups. There were no significant differences between groups on overall flow, *t*(38) = 0.033, *p* = 0.974. Further examination revealed that there were 2 dimensions which were significantly different between groups, with the control scoring higher than the VR experiment group: Clear goals (control *M* = 3.41, VR *M* = 2.79; *t*(38) = 2.08, *p* = 0.045) and Unambiguous feedback (control *M* = 3.61, VR *M* = 3.05; *t*(38) = 2.71, *p* = 0.010). Table 4 shows the means and standard deviations of flow from both groups, as well as the T-scores and p values. Flow was negatively correlated with both state and trait anxiety (Table 5).

**Table 4:** Independent *t*-test results comparing VR and Control group on Flow and dimensions scores

Dimensions	Flow			
	VR	Control	*T* score	*p*
	Mean (SD)	Mean (SD)		
Challenge skill balance	3.63 (0.62)	3.60 (0.78)	− 0.112	0.911
Action awareness	3.85 (0.75)	3.66 (0.68)	− 0.834	0.410
Clear goals	2.79 (0.97)	3.41 (0.94)	2.076	0.045
Unambiguous feedback	3.05 (0.69)	3.61 (0.63)	2.709	0.010
Concentration on task	3.99 (1.00)	3.78 (0.85)	− 0.722	0.475
Sense of control	4.14 (0.76)	4.06 (0.54)	− 0.359	0.721
Loss self-consciousness	4.01 (1.10)	3.78 (0.86)	− 0.761	0.452
Transformation of time	4.09 (0.87)	3.76 (0.79)	− 1.234	0.225
Autotelic experience	4.21 (0.82)	4.14 (0.64)	− 0.320	0.750
Overall flow	33.75 (4.88)	33.80 (4.70)	0.321	0.974

**Table 5:** Correlations for anxiety, flow and presence

	Overall flow	IPQ total	State anxiety pre	Trait anxiety pre	State anxiety post	Trait anxiety post
Overall flow	1					
IPQ total	0.61**	1				
State anxiety pre	− 0.19	− 0.17	1			
Trait anxiety pre	− 0.57**	0.04	0.50**	1		
State anxiety post	− 0.58**	− 0.44	0.25	0.43**	1	
Trait anxiety post	− 0.58**	− 0.09	0.39*	0.87**	0.66**	1

*p < 0.05 (2-tailed), **p < 0.01 (2-tailed)

While the other dimensions were not significantly different, it is interesting to note that the overall trend in mean scores show the VR experiment group scoring higher than the control group.

### State and trait anxiety

Mean scores of STAI showed an overall decrease in state anxiety levels in both groups (VR experiment and control) between pre-activity (*M* = 35.90, *SD* = 7.11; *M* = 30.60, SD = 6.75 respectively) and post-activity (*M* = 28.75, SD = 8.69; *M* = 27.50, SD = 8.13 respectively).

To examine if this reduction was significant, a 2 × 2 mixed factorial ANOVA was conducted on the influence of Time and Group on State Anxiety score. Results revealed that there was a significant main effect of Time on State Anxiety scores overall, (*F*(1, 38) = 11.558, *p* = 0.002, *η*_p_^2^ = 0.233). Noting observed power high at 0.912, and effect size small at 0.233.

There was no significant difference in State-anxiety scores between VR experiment (*M* = 32.33, SD = 1.35) and control (*M* = 29.05, SD = 1.35) groups, *F*(1, 38) = 2.924, *p* = 0.095, *η*_p_^2^ = 0.071, and low power at 0.385. There was no significant Time x Group interaction, *F*(1, 38) = 1.804, *p* = 0.187, *η*_p_^2^ = 0.045, observed power = 0.258.

Simple main effects analysis showed that State-anxiety significantly decreased for the VR experiment group at post-intervention compared to pre-intervention (MD = 7.150, *p* = 0.002), but there were no significant differences in State-anxiety for the control group at post-intervention compared to pre-intervention, (MD = 3.100, *p* = 0.178).

Similarly, for trait anxiety, results indicated an overall decrease in Trait-anxiety scores in both groups (VR experiment and control) between pre-activity (*M* = 39.05, SD = 10.34; *M* = 38.35, SD = 11.08 respectively) and post-activity (*M* = 36.90, SD = 9.40; *M* = 36.65, SD = 12.91 respectively).

To examine if this reduction was significant, a 2 × 2 mixed factorial ANOVA was also conducted on the influence of Time and Group on Trait Anxiety score. Results revealed that there was a significant main effect of Time on Trait Anxiety scores overall, *F*(1, 38) = 4.683, *p* = 0.037, *η*_p_^2^ = 0.110, observed power = 0.559. There was no significant Time × Group interaction, *F*(1, 38) = 0.064, *p* = 0.802, *η*_p_^2^ = 0.002, observed power = 0.057. Also there were no significant simple effects for time when analysed for each group, *p* > 0.05.

## Discussion

The findings of the current study have yielded interesting outcomes. For clarity, they will be discussed in the order of the study’s hypotheses.

1. Equal or greater level of flow will be experienced in the VR experimental condition than in the control condition.

As hypothesised, there was no significant difference in overall levels of flow between groups, suggesting that the level of flow experienced when drawing in VR using Tilt Brush was similar to traditional paper-based drawing. However there are additional benefits of using VR, such as low recurrent costs (e.g. no art supplies), and greater availability, convenience, and accessibility.

Closer examination revealed equivalent levels of flow between groups in all dimensions of flow except in Clear goals and Unambiguous feedback, when the control group performed significantly better. A possible explanation for this difference may be the novelty and lack of mastery of drawing in VR, not only in terms of familiarity with the equipment and technology but also with three-dimensional space, as traditional methods of drawing are usually on two-dimensional planes. This unfamiliarity may have interfered with participants’ perception of their performance, resulting in the view that feedback was unambiguous. According to Jackson (2010), unambiguous feedback is closely related to clear goals as feedback is how one determines that they are heading in the right direction towards the goal. Hence, perceived unclear feedback could have negatively impacted participants’ clarity on goals. In contrast, familiarity with drawing on paper and the use of familiar art materials may have facilitated better flow in these two measures (Clear goals and Unambiguous feedback) due to fewer obstacles to participants immersing themselves in the activity.

2. Equal or greater reduction in state anxiety following the intervention will be reported from the VR experimental condition than from the control condition.

Consistent with prior literature (Tse et al. 2022; Mao et al. 2020), flow was significantly negatively correlated with anxiety, in this case both state and trait anxiety. While trait anxiety did not significantly decline in both groups, this was expected as trait anxiety is more stable as compared to state anxiety, which is more reactive (Spielberger et al. 1983). Consistent with the hypothesis, results support that the VR experiment group had an equivalent reduction in state anxiety as the control group, and post-hoc analysis revealed that the VR experiment group had a significant reduction in state anxiety at post-intervention. However, this should be interpreted with caution as the VR experiment group had a significantly higher level of state anxiety pre-activity compared to the control group. While steps were taken to ensure participants were unaware of their assigned group prior to commencing the study, it may be possible that there was an artificial elevation in state anxiety due to the room set-up (i.e. VR group participants could see the VR equipment upon entering the room), whereas participants in the control group entering the room could see the art supplies.

While it was not hypothesised that trait anxiety would reduce post-activity, it was interesting to observe this in the data. One possibility is that the reduction of state anxiety impacts on the self-report of trait anxiety. Future research could consider replicating the study or using another type of anxiety measure to further understand this observation. With short term and long term effects of anxiety debilitating suffers, it is promising that the overall trend of anxiety scores in this study shows that both groups reduced their levels of anxiety, indicating that drawing in VR and drawing using paper-based materials can both help with anxiety.

3. The level of flow experienced in the VR experimental condition will be positively correlated with the level of presence experienced in the VR experimental condition.

Consistent with the literature, the overall level of flow that participants experienced in this study is positively correlated to the overall level of presence experienced, indicating that the sense of presence is an important factor in experiencing a sense of flow in VR. More specifically, all 3 elements of presence (i.e. Spatial Presence, Involvement, and Experienced Realism) were positively correlated to overall flow. This was consistent with existing research which looked at screen-based gaming (Jin 2011; Nijs et al. 2012; Weibel and Wissmath 2011). Future VR interventions or activities that aim for users to experience flow should consider maximising sense of presence in the design of their programs.

### Limitations

The small sample size of participants was a limitation of this study, which not only impacts the generalisability of results but also on power and effect size of findings. Despite some non-significant results, overall trends (i.e. Flow dimension mean scores, STAI scores) indicate potential for impactful results in favour of the utility of VR in facilitating anxiety-reducing activities. Future research that can accommodate a larger sample size may help to confirm and shed further insights and findings. At points in the study some participants exceeded the clinical cut-offs for State and Trait anxiety as measured using the STAI scores. However sample size prevented any statistical analysis between groups based on the clinical thresholds, limiting the generalisability of findings to other population groups, particularly clinically anxious populations.

Another limitation of the study is the absence of control of potentially confounding factors that could affect anxiety scores, such as caffeine. While the study recorded last caffeine intake of participants the distribution of results did not allow for any statistical between-groups analysis. Caffeine has been shown to have anxiogenic effects (Botella and Parra 2003; Brice and Smith 2002; Winston et al. 2005) and has a half-life ranging from 2.5 to 10 hours with variation affected by biological and lifestyle factors (Evans et al. 2020), therefore recent consumption prior to the study may inflate anxiety scores. It has been suggested that an abstinence of 5–6 hours should eliminate caffeine effects from the body in a healthy population (Grant et al. 2018). Other lifestyle factors that may impact anxiety should be considered as well, such as sleep.

While comfort was not explored in this study, it may be beneficial for future research in this area to explore the invasiveness of wearing the VR headset. Familiarity with the VR technology may impact participants’ capacity to fully immerse themselves in VR, and also the excitement and wow effect experienced by many first-time VR users could artificially impact on their presence, flow and anxiety. Having participants repeat the study could explore the residual effect of VR Tilt Brush and traditional paper-based drawing on anxiety once any wow effect had abrogated.

Mastery has been seen to be negatively associated with anxiety, particularly in the caregiver literature (Mausbach et al. 2012; Roepke et al. 2008; Chan et al. 2018). Thus it is possible that lack of mastery with drawing could affect flow and in turn anxiety. Although the study gave participants time to familiarise themselves with the VR hardware and software, they would not have full mastery of the VR tools for drawing, nor experience of new ways to draw in VR such as with animations and in 3D. Similarly participants may have lacked mastery in traditional paper-based drawing which may have evoked anxiety. It was explained that the drawings created in the study were not to be viewed by the research team nor used in the study, however participants may have felt a pressure to perform. Further, lack of control has been seen to increase anxiety (Hanton and Connaughton 2002), and in this study participants may have felt they were not in control of their environment (either VR Tilt Brush or their physical drawing) and experienced anxiety. Thus future research should also consider examining familiarity, mastery and lack of control as factors.

## Conclusion

The present research demonstrated the efficacy of performing a known anxiety-reducing activity, namely drawing, in VR. The findings indicate that VR-based drawing is as effective as more traditional real-life drawing at reducing anxiety as well as producing equivalent levels of flow. These results provide a basis to implement in VR other activities from clinical treatment programmes, and to pave the way for future explorations into VR-aided counselling and interventions, including inpatient settings or where face-to-face contact is not possible such as during the COVID-19 pandemic. However, larger randomised controlled trials are required before such interventions can be properly endorsed for clinical practice.

## Data Availability

The datasets generated during and/or analysed during the current study are available from the corresponding author on reasonable request.
